# Terrestrial Carbon Cycle Variability

**DOI:** 10.12688/f1000research.8962.1

**Published:** 2016-09-26

**Authors:** Dennis Baldocchi, Youngryel Ryu, Trevor Keenan

**Affiliations:** 1 Department of Environmental Science, Policy and Management, University of California, Berkeley, CA, USA; 2 Department of Landscape Architecture and Rural Systems Engineering, Seoul National University, Seoul, Korea, South; 3 Earth and Environmental Science Division, Lawrence Berkeley National Laboratory, Berkeley, CA, USA

**Keywords:** terrestrial carbon cycle, carbon cycle variability, carbon fluxes

## Abstract

A growing literature is reporting on how the terrestrial carbon cycle is experiencing year-to-year variability because of climate anomalies and trends caused by global change. As CO _2_ concentration records in the atmosphere exceed 50 years and as satellite records reach over 30 years in length, we are becoming better able to address carbon cycle variability and trends. Here we review how variable the carbon cycle is, how large the trends in its gross and net fluxes are, and how well the signal can be separated from noise. We explore mechanisms that explain year-to-year variability and trends by deconstructing the global carbon budget.

The CO _2_ concentration record is detecting a significant increase in the seasonal amplitude between 1958 and now. Inferential methods provide a variety of explanations for this result, but a conclusive attribution remains elusive. Scientists have reported that this trend is a consequence of the greening of the biosphere, stronger northern latitude photosynthesis, more photosynthesis by semi-arid ecosystems, agriculture and the green revolution, tropical temperature anomalies, or increased winter respiration.

At the global scale, variability in the terrestrial carbon cycle can be due to changes in constituent fluxes, gross primary productivity, plant respiration and heterotrophic (microbial) respiration, and losses due to fire, land use change, soil erosion, or harvesting. It remains controversial whether or not there is a significant trend in global primary productivity (due to rising CO _2_, temperature, nitrogen deposition, changing land use, and preponderance of wet and dry regions). The degree to which year-to-year variability in temperature and precipitation anomalies affect global primary productivity also remains uncertain. For perspective, interannual variability in global gross primary productivity is relatively small (on the order of 2 Pg-C y ^-1^) with respect to a large and uncertain background (123 +/- 4 Pg-C y ^-1^), and detected trends in global primary productivity are even smaller (33 Tg-C y ^-2^). Yet residual carbon balance methods infer that the terrestrial biosphere is experiencing a significant and growing carbon sink. Possible explanations for this large and growing net land sink include roles of land use change and greening of the land, regional enhancement of photosynthesis, and down regulation of plant and soil respiration with warming temperatures. Longer time series of variables needed to provide top-down and bottom-up assessments of the carbon cycle are needed to resolve these pressing and unresolved issues regarding how, why, and at what rates gross and net carbon fluxes are changing.

## Introduction

In today’s world, CO _2_ concentrations have risen beyond 400 ppm, a level not experienced over the past 800,000 years ^1^. This rise in atmospheric CO _2_ is mostly due to fossil fuel emissions ^2^ and is largely responsible for a 1.5°C increase in air temperatures over land since the 1880s ^3^. Together, rising CO _2_ concentrations and temperatures are causing the carbon cycle to experience greater year-to-year perturbations and trends than those experienced during the past deglaciation events ^4, 5^.

Today, the constituent fluxes and pools of the terrestrial carbon cycle are widely out of equilibrium from pre-historical conditions owing to human activities. For perspective, atmospheric CO _2_ increased from about 180 to 280 ppm since the last glacial period, adding about 220 Pg-C to the atmosphere over a 10,000-year period ^6^; this pre-industrial change was associated with a positive trend in the net global carbon exchange rate of about 20 Tg-C y ^-1^. In comparison, atmospheric CO _2_ is increasing at a rate of about 4.4 Pg-C y ^-1^, as fossil fuel and cement production release 9 +/- 0.5 Pg-C y ^-1^, land use change releases 0.9 +/- 0.5 Pg-C y ^-1^, and terrestrial ecosystems assimilate 3 +/- 0.5 Pg-C y ^-1^ ^7^.

Net carbon exchange by terrestrial ecosystems is expected to be variable and changing in our warming and CO _2_-enriched world. This expectation is based on the fact that the rates of photosynthesis are tied to CO _2_ and temperature and that respiration is tied to temperature, photosynthesis, and the size of the carbon pools. From first principles, we know that photosynthesis will increase with CO _2_ concentrations in a diminishing returns fashion, defined by Michaelis-Menten reactions associated with the carboxylation reactions between CO _2_ and ribulose bisphosphate. In addition, increased temperatures accelerate kinetic rates of enzyme reactions, thereby increasing mitochondrial respiration of plants and microbes.

On a year-to-year basis, the secular warming of the Earth’s climate is causing different regions of the world to experience episodes of extremes such as wetness, dryness, hot, and cold, which can perturb the fluxes of carbon to and from the plants and soils of those regions ^8– 10^. And, on annual to decadal time scales, changes in land use, phenology, greenness of the biosphere, fires, and nitrogen deposition are introducing additional variability and trends on components of the carbon cycle ^7, 11– 13^.

## How variable is the carbon cycle?

To answer this question with confidence, we have to separate trends and induced variability from natural variability and random sampling errors. We are entering an era where the sampling record is starting to be long enough to separate signal from noise. We have a 50-year record of CO _2_ concentrations in the atmosphere, providing a top-down constraint on carbon cycle variability ^7^, and we have a 30-year satellite record, giving us bottom-up information on spatial/temporal variability of the greening of the land ^13^. Consequently, there has been growing activity to quantify and understand variability of the carbon cycle based on these longer global-scale records. The objective of this review is to survey key literature on the variability of the terrestrial carbon cycle at the global scale published over the past 4+ years (2012 into 2016).

To assess any attribution in the variability of the terrestrial carbon cycle, one must consider the degree of differential and induced modulations of its three major carbon pools. The vegetation, soil, and atmosphere carbon pools have different sizes, different turnover times, and different responses to environmental perturbations, like light, CO _2_ concentration, temperature, and soil moisture ^14^. In other words, there is a disequilibrium between the gains and losses of carbon to and from the plant and soil pools, which can partly be explained by the relatively fast way CO _2_ enters the biosphere via photosynthesis and the relatively slow way it leaves via plant, root, and soil respiration. Consequently, these carbon pools have different susceptibility to anomalous weather and climate variability at global and regional scales. Furthermore, the variability of carbon fluxes is dependent upon changes in such ecological factors as plant functional type, leaf area index, time since disturbance and stand age, nitrogen loading, the intensity and frequency of fires, soil erosion, and transport as dissolved carbon.

Given the superposition of natural (by weather and fire) and human-induced variability (by climate change, increasing CO _2_, changing land use, changing forest age distributions, pollution, and nitrogen deposition) on the carbon cycle ^8^, can we detect signals or responses among simultaneous variation of numerous drivers? Secondly, are the short-term changes in carbon pools large enough to detect with current observation systems? In assessing if variability in the carbon cycle is occurring, we must consider the detection limit and sampling errors of these systems as they affect how precise or accurate they are.

**Figure 1.**
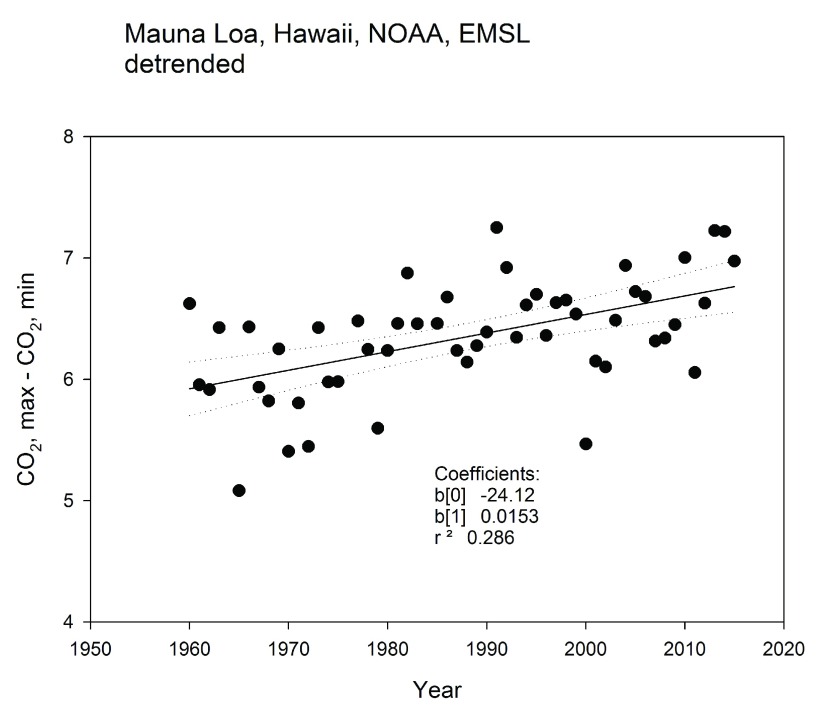
Variation in the amplitude of CO _2_ at Mauna Loa, Hawaii. Data are courtesy of NOAA Global Monitoring Division ( http://www.esrl.noaa.gov/gmd/), Ed Dlugokencky and Pieter Tans, NOAA/ESRL ( www.esrl.noaa.gov/gmd/ccgg/trends/).

One group of scientists has reported that the amplitude of the seasonal swing in atmospheric CO _2_ is growing ^4, 15, 16^. To illustrate this point, we show publicly available data from the long-term monitoring station at Mauna Loa, Hawaii ( Figure 1). This figure shows that the magnitude of the difference between the maximum and minimum values of CO _2_ concentration for each year, after the time series was detrended with a moving filter, has increased by nearly 15% over 55 years. When the global fields of CO _2_, from a network of CO _2_ sampling sites, are considered, it has been deduced that the annual global carbon uptake doubled from 2.46 to 5.06 Pg-C y ^-1^ between 1960 and 2010, a rate of 52 Tg-C y ^-2^ ^5^.

What is the explanation for this temporal increase in CO _2_ amplitude? The answers are manifold. Scientists have reported that this trend is a consequence of the greening of the biosphere ^13^, stronger northern latitude photosynthesis ^4, 15^, more photosynthesis by semi-arid ecosystems ^17, 18^, agriculture and the green revolution ^16, 19^, tropical temperature anomalies ^20^, or increased winter respiration ^21^.

Evidence for the greening of the terrestrial biosphere is provided by an analysis of satellite remote sensing data. The scientists report that seasonally integrated leaf area index is increasing across a quarter to one-half of the planet’s vegetated area, mostly to the CO _2_ fertilization effect ^13^. Browning is detected too, but on only 4% of the vegetated land area.

Other scientists point to the northern latitudes, home of the world’s boreal forests, as the locale for a growing carbon sink. Graven *et al*. ^4^ reported that a 30 to 60% change has occurred in the carbon exchange of boreal forests. Forkel *et al*. ^15^ lend support to this hypothesis by reporting that “the latitudinal gradient of the increasing CO _2_ amplitude is mainly driven by positive trends in photosynthetic carbon uptake caused by recent climate change and mediated by changing vegetation cover in northern ecosystems”. Others claim winter conditions may have a strong influence on year-to-year variations in the growth of the CO _2_ concentration amplitude. Yu *et al*. ^21^ argue that warmer winters have less snow, which causes soil temperatures to be colder, reducing winter respiration. They conclude that this mechanism explains 25% of the enhancement of the carbon sink across boreal forests.

In addition, not all scientists agree on whether the activity of boreal forests explains the variability of the terrestrial carbon cycle. One team ^20^ reported that 50% of the interannual variation of the CO _2_ growth rate, between 1959 and 2011, was associated with tropical air temperature; a 1°C anomaly in tropical air temperature corresponded with a 3.5 +/- 0.6 Pg-C y ^-1^ anomaly in the CO _2_ growth rate. Others attribute the trends in the global carbon sink to the growth of semi-arid vegetation ^17, 18^; semi-arid ecosystems are experiencing a 0.04 Pg-C y ^-2^ trend in their carbon sink, which is about 57% of the global trend. Two other groups point to the role of agriculture as a modulating factor of the global CO _2_ concentration record. Gray *et al*. ^16^ and Zeng *et al*. ^19^ attribute a significant part of the increase in the CO _2_ seasonal amplitude (17 to 25%) to the agricultural green revolution; cereal production in the northern hemisphere increased by 240 percent over the 47-year period between 1961 and 2008, thereby increasing the net carbon uptake of crops by 0.33 Pg-C.

## Deconstructing the global carbon cycle

To better understand how and why the terrestrial carbon cycle is experiencing variability, let’s examine the potential for modulation and or trends in the distinct plant and soil carbon pools.

### Global gross primary productivity

The carbon cycle starts with the input of carbon through gross primary productivity. What is the value of global gross primary productivity? There is no single sensor or method to perform this assessment perfectly ^22^. Consequently, scientists are using networks of carbon flux measurements and meteorological stations with remote sensing and machine learning techniques to produce spatially resolved flux maps on monthly scales that can be summed to produce a global estimate ^23, 24^. These empirically based, machine learning estimates of global gross primary productivity range from between 119 +/- 6 and 123 +/- 4 Pg-C y ^-1^.

Given this uncertainty, how variable is global gross primary production on a temporal basis? One could assume that, to a first order, global photosynthesis may experience little year-to-year variation at the global scale because it is primarily a function of solar radiation, and the solar constant is relatively stable at 1360 +/- 0.5 W m ^-2^ ^25^.

Yet other studies beg to differ and report significant variation and trends in global photosynthesis. For example, a synthesis of 10 modeling approaches for assessing global gross primary productivity showed a range of between 112 and 169 Pg-C y ^-1^, a mean of 138 +/- 17 Pg-C y ^-1^, interannual variability on the order of 2.64 +/- 1.12 Pg-C y ^-1^, and a sensitivity of the long-term trend of 33 +/- 23 Tg-C y ^-2^ ^26, 27^.

Temperature is bound to have different effects on photosynthesis on a regional basis. Piao *et al*. ^27^ report that global gross primary productivity has a negative relationship with temperature in the tropics and a positive relationship with temperature in the boreal regions owing to a longer growing season. At the global scale, they found that interannual variability in gross primary productivity is not correlated to its global temperature anomalies. Year-to-year variations in air temperature can also affect photosynthesis indirectly by its impact on phenology and the length of the growing season ^28, 29^. Emergent properties like the acclimation of photosynthesis to temperature must be considered too ^30^ when contemplating the change in gross primary productivity to warmer temperatures.

Regional drought can limit photosynthesis by causing physiological stress ^10, 31^. Integrating these drought effects globally, carbon cycle models reveal that average global gross primary productivity increases by 4.1 +/- 2 Pg-C y ^-1^ per 100 mm of precipitation ^27^. In humid tropical regions, moderate drought can enhance photosynthesis since it leads to reduced cloud cover and produces more incident sunlight ^32^.

Extreme climate events may have a disproportionate effect on global gross primary productivity. One study produced a 30-year record of the variability of global gross primary productivity using remote sensing data from a global flux network and a set of carbon cycle and dynamic vegetation models ^33^. The authors reported that a few extreme events explain most of the interannual variability in global gross primary productivity; 78% of the global anomaly in gross primary productivity is associated with 200 extreme events in the tenth percentile ^33^.

The examination in temporal trends in global gross primary productivity, inferred from long-term satellite records, has the potential to detect if the terrestrial biosphere is experiencing a response to rising CO _2_ on global carbon assimilation. A new study ^34^ assessed the large divergence between satellite and Earth system models regarding the CO _2_ fertilization effect on the carbon cycle. The authors found that net primary productivity derived from satellites increased by 2.8% from 1982 to 2011. In contrast, estimates of global net primary productivity, the difference between gross primary productivity and autotrophic respiration, derived by Earth system models increased by 7.6% over 30 years. Smith *et al*. conclude that Earth system models may be oversensitive to CO _2_ effect if the satellite inferred method is correctly sampling the response of the biosphere to a secular increase in CO _2_. A comment on the Smith *et al*. paper ^35^, however, points out that the satellite estimates used explicitly exclude the direct effect of CO _2_ on gross primary production and suggests that the opposite conclusion is a more appropriate interpretation of the Smith *et al*. data: that remote sensing estimates likely under-predict the response of gross primary production to CO _2_.

Another set of studies infer that long-term trends in global primary productivity may be emerging owing to enhancement by elevated carbon dioxide and fertilization by nitrogen deposition. Schimel *et al*. ^12^ asked, “what is the effect of rising CO _2_ concentration on the carbon cycle?” They argued that theory predicts that the enhancement of photosynthesis by CO _2_ should have a tropical maximum. They evaluated data over the 2000 to 2010 timeframe using nine process models. They concluded that there was significant tropical uptake. Their results suggest that up to 60% of the present-day terrestrial sink is caused by increasing atmospheric CO _2_. As for hard numbers, they report that the best estimate of the tropical + southern CO _2_ enhancement effect was a sink of -1.4 ± 0.4 Pg-C y ^−1^; negative values indicate a loss of carbon from the atmosphere and a gain by the biosphere. Such debates highlight the large unknowns that remain regarding how global photosynthesis is responding to changes in atmospheric CO _2_.

Land use change can affect regional and global photosynthesis both positively and negatively. Land use change and rates of land use change affect the extent or direction of the change (deforestation or reforestation) in the green land area and the number of leaves intercepting photons ^13^.

**Figure 2.**
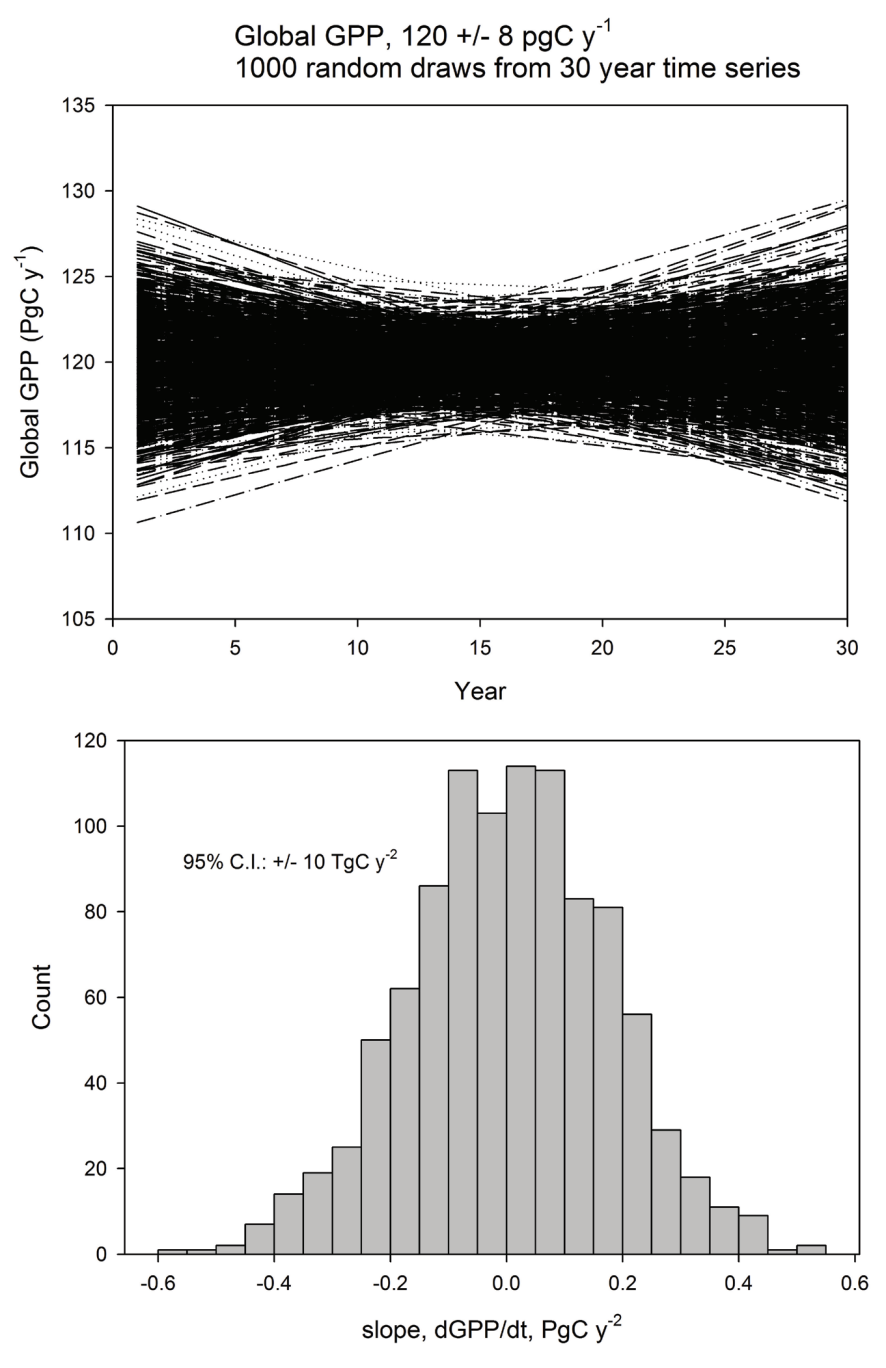
**a**) Trends in global gross primary production (GPP) of a 30-year-long time series derived from a population of random numbers that were sampled from a Gaussian distribution of +/- 4 Pg-C y ^-1^ 1000 times; **b**) histogram and 95% confidence interval of the slopes derived from the populations of slopes computed in part **a**). The standard deviation is 0.0856 Pg-C y ^-2^ and the 95% confidence interval is +/-5.3 Tg-C y ^-2^.

Questions remain as to the certainty of inferring small changes in global photosynthesis with confidence given the degree of measurement and sampling errors that are associated with upscaling photosynthesis to the global scale. One can estimate the 95% confidence interval that random trends in global gross primary productivity must exceed. We assumed that the uncertainty about global gross primary productivity with an empirical artificial neural network method ^23^ is on the order of +/- 4 Pg-C y ^-1^. Next, we drew a random population (n=1000) of a 30-year time series, about this error, using a Gaussian random number generator. We then fit linear regressions through each of these randomly probable trends and produced a histogram of their slopes ( Figure 2). We found that trends in global gross primary productivity must have a slope exceeding +/- 5.3 Tg-C y ^-2^ to exceed the 95% confidence interval of the randomly sampled trends. If we assume the interannual uncertainty in global gross photosynthesis is on the order of 2.64 Pg-C y ^-1^, as shown, the 95% confidence interval of the slope is bound within +/- 3.4 Tg-C y ^-2^.

Clearly, more work and longer datasets are needed to resolve the contrasting conclusions derived from the satellite-based inferences and the model upscaling methods of global gross photosynthesis. New efforts are underway to produce independent estimates and constraints on global photosynthesis with sun-induced fluorescence ^36^ and to expand existing global networks of land-atmosphere CO _2_ exchange, which should shed new light on this question ^37^.

### Global respiration and oxidation losses

Photosynthesis is offset by plant and root respiration; these respiratory processes scale with temperature, soil moisture, and the physiological activity of plants. Trends or climatic anomalies in any of these biophysical variables have the potential to cause variations in plant respiration. How such anomalies scale globally depends on how well wet and dry and cool and hot climate anomalies average out globally and the degree to which climate extremes may push non-linear responses ^9^. Other losses of plant material can arise through fire ^38^. Variations in carbon losses will be a function of fire area, intensity, and frequency ^38– 40^. Regarding carbon lost from the soil, trends or anomalies in temperature, soil moisture, water table, and presence and absence of snow can modulate these effluxes ^21^. The potential for large carbon losses at regional scales can occur with the drying and thawing of the permafrost and drying of tropical peat forests.

### Other losses and gains: disturbances and regrowth

On land, tropical forests are being lost, temperate forests are being regenerated, and semi-arid ecosystems burn periodically ^7, 38^. Years with *El Niño* have led to large fire emissions from southeast Asia, as drought allowed loggers into the normally flooded peat forests ^39^. Other losses of soil carbon can be attributed to erosion (0.3 to 1.0 Pg-C y ^-1^) ^41^ and transport to the oceans in the form of dissolved inorganic and organic carbon ^42^.

### Net biome productivity

Net biome productivity (NBP) is the balance between gross primary productivity and losses attributed to autotrophic and heterotrophic respiration and disturbance/harvesting losses. The final question we must ask is how do all the potential gains and losses to the global terrestrial carbon cycle add up? A new study has brought new light to this sink by examining the net land greenhouse gas fluxes (associated with land plants, animals, and microbes) using a range of bottom-up and top-down modeling approaches that considered land use change, rising CO _2_, N deposition, and climate variability in tandem ^11^. They reported that the global net carbon sink increased fourfold between the 1980s (when it ranged between -1.2 and -1.4 Pg-C y ^-1^) and the 2000s (when it ranged between -5.3 and -5.8 Pg-C y ^-1^).

## Concluding remarks

The variability of the global carbon cycle is changing, as the amplitude of seasonal CO _2_ concentration and the net land sink are increasing. The causes of these trends in the global carbon cycle remain uncertain and a challenge for future research.

In this review, we show that different analyses have different explanations for why the seasonal CO _2_ amplitude and land sink are increasing. We advise investigators of future studies to test whether or not reported trends are significantly different from random errors associated with the uncertainties in modeling and measuring gross and net carbon fluxes.

Photosynthesis is the starting point of the carbon cycle. Yet, despite decades of research, our ability to produce an estimate of global photosynthesis with narrow confidence intervals and high accuracy remains elusive. Only with more accurate estimates of photosynthesis will we be able to better resolve and understand differences between data-driven and process models and to better simulate responses between net and gross carbon fluxes to temperature, precipitation, CO _2_, and nitrogen.

The response of global photosynthesis to CO _2_ fertilization remains unresolved, as the variations in the assimilation fluxes are small relative to the uncertainties of the drivers and information systems, using bottom-up and top-down methods. Indirect effects can be important too. For example, lengthening growing seasons can increase gross primary productivity on regional scales ^43^. Vegetative regrowth may be offsetting losses of carbon by preceding fires. Thawing of the permafrost may expose and release vast stores of carbon that have been decoupled for millennia, while warming northlands are getting greener. Many experiments show acclimation effects of respiration to temperature ^44^. This is a process that is not well considered by models, though some are starting to consider this factor ^30^.

In closing, we need to continue the collection of long-term and multi-faceted products that are used to assess the global carbon cycle. Additional data from new satellites and longer time series from eddy covariance and CO _2_ concentration networks will reduce uncertainty in sampling and modeling and improve our ability to answer the questions associated with carbon cycle variability and trends.

## References

[1] Augustin L Barbante C Barnes PR: Eight glacial cycles from an Antarctic ice core. *Nature.* 2004;429(6992):623–8. 10.1038/nature02599 15190344

[2] Francey RJ Allison CE Etheridge DM: A 1000-year high precision record of delta ^13^C in atmospheric CO _2_. *Tellus B.* 1999;51(2):170–93. 10.1034/j.1600-0889.1999.t01-1-00005.x

[3] Blunden J Arndt DS: State of the Climate in 2014. *Bull Amer Meteor Soc.* 2015;96(7):ES1–ES32. 10.1175/2015BAMSStateoftheClimate.1

[4] Graven HD Keeling RF Piper SC: Enhanced seasonal exchange of CO _2_ by northern ecosystems since 1960. *Science.* 2013;341(6150):1085–9. 10.1126/science.1239207 23929948

[5] Ballantyne AP Alden CB Miller JB: Increase in observed net carbon dioxide uptake by land and oceans during the past 50 years. *Nature.* 2012;488(7409):70–2. 10.1038/nature11299 22859203

[6] Petit JR Jouzel J Raynaud D: Climate and atmospheric history of the past 420,000 years from the Vostok ice core, Antarctica. *Nature.* 1999;399:429–36. 10.1038/20859

[7] Le Quéré C Moriarty R Andrew RM: Global Carbon Budget 2015. *Earth Syst Sci Data.* 2015;7:349–96. 10.5194/essd-7-349-2015

[8] Reichstein M Bahn M Ciais P: Climate extremes and the carbon cycle. *Nature.* 2013;500(7462):287–95. 10.1038/nature12350 23955228

[9] Frank D Reichstein M Bahn M: Effects of climate extremes on the terrestrial carbon cycle: concepts, processes and potential future impacts. *Glob Chang Biol.* 2015;21(8):2861–80. 10.1111/gcb.12916 25752680 PMC4676934

[10] Wolf S Keenan TF Fisher JB: Warm spring reduced carbon cycle impact of the 2012 US summer drought. *Proc Natl Acad Sci U S A.* 2016;113(21):5880–5. 10.1073/pnas.1519620113 27114518 PMC4889356

[11] Tian H Lu C Ciais P: The terrestrial biosphere as a net source of greenhouse gases to the atmosphere. *Nature.* 2016;531(7593):225–8. 10.1038/nature16946 26961656

[12] Schimel D Stephens BB Fisher JB: Effect of increasing CO _2_ on the terrestrial carbon cycle. *Proc Natl Acad Sci U S A.* 2015;112(2):436–41. 10.1073/pnas.1407302112 25548156 PMC4299228

[13] Zhu Z Piao S Myneni RB: Greening of the Earth and its drivers. *Nat Clim Chang.*Advance online publication.2016;6(8):791–795. 10.1038/nclimate3004

[14] Bloom AA Exbrayat JF van der Velde IR: The decadal state of the terrestrial carbon cycle: Global retrievals of terrestrial carbon allocation, pools, and residence times. *Proc Natl Acad Sci U S A.* 2016;113(5):1285–90. 10.1073/pnas.1515160113 26787856 PMC4747711

[15] Forkel M Carvalhais N Rödenbeck C: Enhanced seasonal CO _2_ exchange caused by amplified plant productivity in northern ecosystems. *Science.* 2016;351(6274):696–9. 10.1126/science.aac4971 26797146

[16] Gray JM Frolking S Kort EA: Direct human influence on atmospheric CO _2_ seasonality from increased cropland productivity. *Nature.* 2014;515(7527):398–401. 10.1038/nature13957 25409830

[17] Ahlström A Raupach MR Schurgers G: Carbon cycle. The dominant role of semi-arid ecosystems in the trend and variability of the land CO _2_ sink. *Science.* 2015;348(6237):895–9. 10.1126/science.aaa1668 25999504

[18] Poulter B Frank D Ciais P: Contribution of semi-arid ecosystems to interannual variability of the global carbon cycle. *Nature.* 2014;509(7502):600–3. 10.1038/nature13376 24847888

[19] Zeng N Zhao F Collatz GJ: Agricultural Green Revolution as a driver of increasing atmospheric CO _2_ seasonal amplitude. *Nature.* 2014;515(7527):394–7. 10.1038/nature13893 25409829

[20] Wang W Ciais P Nemani RR: Variations in atmospheric CO _2_ growth rates coupled with tropical temperature. *Proc Natl Acad Sci U S A.* 2013;110(32):13061–6. 10.1073/pnas.1219683110 23884654 PMC3740858

[21] Yu Z Wang J Liu S: Decrease in winter respiration explains 25% of the annual northern forest carbon sink enhancement over the last 30 years. *Glob Ecol Biogeogr.* 2016;25(5):586–95. 10.1111/geb.12441

[22] Canadell JG Mooney HA Baldocchi DD: Commentary: Carbon Metabolism of the Terrestrial Biosphere: A Multitechnique Approach for Improved Understanding. *Ecosystems.* 2000;3(2):115–30. 10.1007/s100210000014

[23] Beer C Reichstein M Tomelleri E: Terrestrial gross carbon dioxide uptake: global distribution and covariation with climate. *Science.* 2010;329(5993):834–8. 10.1126/science.1184984 20603496

[24] Jung M Reichstein M Margolis HA: Global patterns of land-atmosphere fluxes of carbon dioxide, latent heat, and sensible heat derived from eddy covariance, satellite, and meteorological observations. *J Geophys Res.* 2011;116(G3). 10.1029/2010JG001566

[25] Kopp G Lean JL: A new, lower value of total solar irradiance: Evidence and climate significance. *Geophys Res Lett.* 2011;38(1):n/a–n/a. 10.1029/2010GL045777

[26] Anav A Friedlingstein P Beer C: Spatiotemporal patterns of terrestrial gross primary production: A review. *Rev Geophys.* 2015;53(3):785–818. 10.1002/2015RG000483

[27] Piao S Sitch S Ciais P: Evaluation of terrestrial carbon cycle models for their response to climate variability and to CO _2_ trends. *Glob Chang Biol.* 2013;19(7):2117–32. 10.1111/gcb.12187 23504870

[28] Migliavacca M Reichstein M Richardson AD: Influence of physiological phenology on the seasonal pattern of ecosystem respiration in deciduous forests. *Glob Chang Biol.* 2015;21(1):363–76. 10.1111/gcb.12671 24990223

[29] Richardson AD Keenan TF Migliavacca M: Climate change, phenology, and phenological control of vegetation feedbacks to the climate system. *Agric For Meteorol.* 2013;169:156–73. 10.1016/j.agrformet.2012.09.012

[30] Lombardozzi DL Bonan GB Smith NG: Temperature acclimation of photosynthesis and respiration: A key uncertainty in the carbon cycle-climate feedback. *Geophys Res Lett.* 2015;42(20):8624–31. 10.1002/2015GL065934

[31] Reichstein M Ciais P Papale D: Reduction of ecosystem productivity and respiration during the European summer 2003 climate anomaly: A joint flux tower, remote sensing and modelling analysis. *Global Change Biol.* 2007;13(3):634–51. 10.1111/j.1365-2486.2006.01224.x

[32] Hirano T Segah H Harada T: Carbon dioxide balance of a tropical peat swamp forest in Kalimantan, Indonesia. *Global Change Biol.* 2007;13(2):412–25. 10.1111/j.1365-2486.2006.01301.x

[33] Zscheischler J Mahecha MD Buttlar J von: A few extreme events dominate global interannual variability in gross primary production. *Environ Res Lett.* 2014;9(3):35001 10.1088/1748-9326/9/3/035001

[34] Kolby Smith W Reed SC Cleveland CC: Large divergence of satellite and Earth system model estimates of global terrestrial CO _2_ fertilization. *Nature Climate Change.* 2015;6(3):306–10. 10.1038/nclimate2879

[35] De Kauwe MG Keenan TF Medlyn BE: Satellite based estimates underestimate the effect of CO _2_ fertilisation on NPP. *Nature Clim Change.*(in review).

[36] Frankenberg C O'Dell C Berry J: Prospects for chlorophyll fluorescence remote sensing from the Orbiting Carbon Observatory-2. *Remote Sens Environ.* 2014;147:1–12. 10.1016/j.rse.2014.02.007

[37] Baldocchi D: Measuring fluxes of trace gases and energy between ecosystems and the atmosphere - the state and future of the eddy covariance method. *Glob Chang Biol.* 2014;20(12):3600–9. 10.1111/gcb.12649 24890749

[38] Randerson JT Chen Y van der Werf GR: Global burned area and biomass burning emissions from small fires. *J Geophys Res.* 2012;117(G4):n/a–n/a. 10.1029/2012JG002128

[39] Turetsky MR Benscoter B Page S: Global vulnerability of peatlands to fire and carbon loss. *Nature Geosci.* 2014;8:11–4. 10.1038/ngeo2325

[40] Moritz MA Batllori E Bradstock RA: Learning to coexist with wildfire. *Nature.* 2014;515(7525):58–66. 10.1038/nature13946 25373675

[41] Chappell A Baldock J Sanderman J: The global significance of omitting soil erosion from soil organic carbon cycling schemes. *Nature Clim Change.* 2016;6:187–191. 10.1038/nclimate2829

[42] Butman D Raymond PA: Significant efflux of carbon dioxide from streams and rivers in the United States. *Nature Geosci.* 2011;4:839–42. 10.1038/ngeo1294

[43] Keenan TF Darby B Felts E: Tracking forest phenology and seasonal physiology using digital repeat photography: A critical assessment. *Ecol Appl.* 2014;24(6):1478–89. 10.1890/13-0652.1 29160668

[44] Way DA Yamori W: Thermal acclimation of photosynthesis: on the importance of adjusting our definitions and accounting for thermal acclimation of respiration. *Photosynth Res.* 2014;119(1–2):89–100. 10.1007/s11120-013-9873-7 23812760

